# Physiological performance and inflammatory markers as indicators of complications after oesophageal cancer surgery

**DOI:** 10.1002/bjs5.50328

**Published:** 2020-08-04

**Authors:** A. G. M. T. Powell, C. Eley, T. Abdelrahman, A. H. Coxon, C. Chin, I. Appadurai, R. Davies, D. M. Bailey, W. G. Lewis

**Affiliations:** ^1^ Division of Cancer and Genetics Cardiff University Cardiff UK; ^2^ Departments of Surgery Cardiff UK; ^3^ Anaesthesia University Hospital of Wales Cardiff UK; ^4^ Neurovascular Research Laboratory, Faculty of Life Sciences and Education University of South Wales Pontypridd UK

## Abstract

**Background:**

The extent to which physiological factors influence outcome following oesophageal cancer surgery is poorly understood. This study aimed to evaluate the extent to which cardiorespiratory fitness and selected metabolic factors predicted complications after surgery for carcinoma.

**Methods:**

Two hundred and twenty‐five consecutive patients underwent preoperative cardiopulmonary exercise testing to determine peak oxygen uptake ($\dot{V}$ o_2peak_), anaerobic threshold and the ventilatory equivalent for carbon dioxide ($\dot{V}$ e/$\dot{V}$ co_2_). Cephalic venous blood was assayed for serum C‐reactive protein (CRP) and albumin levels, and a full blood count was done. The primary outcome measure was the Morbidity Severity Score (MSS).

**Results:**

One hundred and ninety‐eight patients had anatomical resection. A high MSS (Clavien–Dindo grade III or above) was found in 48 patients (24·2 per cent) and was related to an increased CRP concentration (area under the receiver operating characteristic (ROC) curve (AUC) 0·62, *P* = 0·001) and lower $\dot{V}$ o_2peak_ (AUC 0·36, *P* = 0·003). Dichotomization of CRP levels (above 10 mg/l) and $\dot{V}$ o_2peak_ (below 18·6 ml per kg per min) yielded adjusted odds ratios (ORs) for a high MSS of 2·86 (*P* = 0·025) and 2·92 (*P* = 0·002) respectively. Compared with a cohort with a low Combined Inflammatory and Physiology Score (CIPS), the OR was 1·70 (95 per cent c.i. 0·85 to 3·39) for intermediate and 27·47 (3·12 to 241·69) for high CIPS (*P* < 0·001).

**Conclusion:**

CRP and $\dot{V}$ o_2peak_ were independently associated with major complications after potentially curative oesophagectomy for cancer. A composite risk score identified a group of patients with a high risk of developing complications.

## Graphical abstract

C‐reactive protein concentration and peak oxygen uptake are collective independent risk factors that can account for over half of major morbidity following oesophagectomy for oesophageal cancer. A composite risk score, the Combined Inflammatory and Physiology Score (CIPS) (range 0–2), predicted major morbidity (17·9 per cent in patients with a CIPS of 0 *versus* 86 per cent in those with a CIPS of 2; *P* < 0·001).

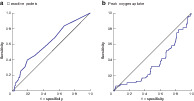

Score identifies high risk group

## Abstract (es)

**Antecedentes:**

El grado en el que los factores fisiológicos influyen en el resultado tras la cirugía del cáncer de esófago no se conoce bien. Este estudio tuvo como objetivo evaluar en qué medida el estado cardiorrespiratorio y los factores metabólicos seleccionados predecían complicaciones después de cirugía por cáncer.

**Métodos:**

Pacientes consecutivos fueron sometidos a una prueba de ejercicio cardiopulmonar preoperatoria para determinar el consumo pico de oxígeno (*peak oxygen uptake*, $\dot{V}$O_2Peak_), el umbral anaeróbico (*anaerobic threshold*, AT) y el equivalente ventilatorio de dióxido de carbono (*ventilatory equivalent for carbon dioxide*, $\dot{V}$_E_/$\dot{V}$_CO2_). Se extrajo sangre de la vena cefálica para analizar la proteína C reactiva (*C‐reactive protein*, CRP) sérica, albumina y hemograma completo. La medida de resultado primario fue la puntuación de la gravedad de la morbilidad (*Morbidity Severity Score*, MSS).

**Resultados:**

Se observó MSS (Clavien‐Dindo > 2) en 33 (17,7%) pacientes, relacionándose con CRP elevada (AUC 0,69, *P* = 0,001) y *V* ˙O_2Peak_ baja (AUC 0,33, *P* = 0,003). La dicotomización de la CRP (por encima de 10 mg/L) y *V* ˙O_2Peak_ (por debajo de 18,6 mL/kg/min) se asociada a una razón de oportunidades (*odds rati*o, OR) de 4,01 (*P* = 0,002) y 3,74 (*P* = 0,002) para MSS y CD > 2, respectivamente. En comparación con la cohorte con una puntuación combinada inflamatoria y fisiológica (*Combined Inflammatory and Physiology Score*, CIPS) baja, el OR fue de 1,70 (i.c. del 95% 0,85‐3,39) para una CIPS intermedia y de 27,47 (3,12‐241,69, *P* < 0,001) para CIPS elevada.

**Conclusión:**

CRP y *V* ˙O_2Peak_ se asociaron de forma independiente con complicaciones mayores tras esofaguectomía potencialmente curativa por cáncer. Una puntuación combinada de riesgo identificó a un grupo de pacientes con un riesgo elevado de desarrollar complicaciones.

## Introduction

Oesophagectomy remains the primary therapeutic modality for potentially curative treatment for patients with oesophageal cancer. Despite recent advances in anaesthesia and critical care, it continues to be associated with considerable morbidity. The 2018 UK National Oesophago‐Gastric Cancer Audit^1^ reported postoperative morbidity and mortality rates of 50 and 1·6 per cent respectively. A number of approaches to risk prediction have been proposed in addition to clinical judgement: objective scoring systems such as Portsmouth POSSUM (P‐POSSUM)^2^, Oesophagogastric POSSUM (O‐POSSUM)^3^, ASA physical status, Charlson Co‐morbidity Index, serum biomarkers^4^, measures of cardiac function^5^ and shuttle walk tests^6^.

Cardiopulmonary exercise testing (CPET) is a non‐invasive and dynamic procedure that allows an individual's cardiopulmonary fitness to be measured accurately^7^. CPET, in particular an anaerobic threshold of less than 11 ml per kg per min, has been reported to predict postoperative morbidity and mortality in patients undergoing major abdominal surgery^8^, ^9^, ^10^. While established in cardiothoracic surgery^11^, the application of CPET in the setting of oesophageal cancer surgery is limited^10^, ^12^.

Inflammation is a hallmark of cancer^13^, and can be measured using cellular (neutrophils, lymphocytes, platelets) and humoral (C‐reactive protein (CRP) and albumin) component measurements^3^. Despite emerging evidence that the systemic inflammatory response (SIR) is associated with postoperative morbidity in colorectal cancer^4^, ^14^, confirmatory evidence in oesophageal cancer is sparse^15^.

In view of the above, the present study examined the extent to which select measures of cardiorespiratory fitness and metabolic markers of inflammation can predict clinical outcome in patients with oesophageal cancer scheduled for elective surgery. The hypothesis was that impaired cardiorespiratory fitness and raised CRP levels would predict patient morbidity. The primary outcome measure was the postoperative Morbidity Severity Score (MSS).

## Methods

Ethical approval was sought from the regional ethics committee, but a formal application was deemed unnecessary because the study was considered to be a service evaluation of consecutively recruited patients, for whom consent had already been provided.

### Patients

A single cohort of patients diagnosed with oesophageal cancer between 1 January 2009 and 31 March 2020 was developed; it included patients with radiological TNM stage I–IV, deemed fit for treatment with curative intent. Patients were staged using CT, endoscopic ultrasonography, CT–PET, and staging laparoscopy as considered appropriate, using patient algorithms for oesophageal cancer as described previously^16^, ^17^. The majority of patients received two cycles of cisplatin 80 mg/m^2^ and 5‐fluorouracil (5‐FU) 1000 mg/m^2^ for 4 days. A minority received three cycles of epirubicin 50 mg/m^2^, cisplatin 60 mg/m^2^, and 5‐FU 200 mg/m^2^ or capecitabine 625 mg/m^2^.

### Surgical intervention

The standard operative approach was open subtotal transthoracic oesophagectomy (TTO), as described by Lewis^18^ and Tanner^19^. Transhiatal oesophagectomy (THO), as described by Orringer^20^, was used selectively in patients with adenocarcinoma of the lower third of the oesophagus who had significant cardiorespiratory co‐morbidity, cT1–3 N0 disease. Laparoscopically assisted surgery was used for a small number of patients.

### Clinicopathological characteristics

Tumours were staged using the seventh edition of the AJCC/UICC TNM staging system. Pathological factors were recorded from pathology reports issued at the time of surgery using the AJCC/UICC TNM staging system (seventh edition), and included tumour differentiation, number of lymph nodes with and without metastasis, and margin status.

Routine laboratory measurements of haemoglobin, whole white cell count, neutrophil count, lymphocyte count, platelet counts, CRP and albumin on the day before surgery were recorded. Derivate measurements of systemic inflammation consisted of the neutrophil/lymphocyte ratio (NLR) and platelet/lymphocyte ratio (PLR)^21^, ^22^.

### Cardiopulmonary exercise testing

CPET followed American Thoracic Society/American College of Chest Physicians recommendations^11^. All patients performed a symptom‐limited CPET conducted on an electromagnetically braked cycle ergometer; this comprised a 2–3‐min rest phase (to allow gas exchange variables to stabilize), 3 min of unloaded cycling, then a ramped incremental protocol until volitional termination, and a 2–5‐min recovery period. Ventilation and gas exchange was measured with a Medgraphics Ultima™ metabolic cart (Medical Graphics, St Paul, Minnesota, USA) with Breezesuite™ (Medical Graphics) and Welch Allyn® (Welch Allyn, New York, USA) software, as described previously^12^.

Heart rate monitoring, BP, pulse oximetry and 12‐lead ECG were done throughout. The ramp gradient was set to 10–20 W based on the predicted $\dot{V}$ o_2peak_ from the age, weight, height and sex of the patient, to produce an exercise test of 8–12 min in duration^23^. Before each test, the CPET equipment was calibrated against reference gases. The anaerobic threshold was determined using the V‐slope method and confirmed by changes in ventilatory efficiency for oxygen ($\dot{V}$ e/$\dot{V}$ co_2_) and the end‐tidal partial pressure of oxygen (*P*eto_2_)^23^. The anaerobic threshold was validated independently by two experienced observers. $\dot{V}$ o_2peak_ was the highest $\dot{V}$ o_2_ achieved during the final 30 s of the test. The $\dot{V}$ e/$\dot{V}$ co_2_ slope was measured at the anaerobic threshold. Test termination criteria included: request of patient, volitional fatigue, chest or leg pain, or ECG abnormalities determined by the consultant anaesthetist. Multidisciplinary discussion and stratification of individual patient risk informed decisions regarding the planned postoperative level of care and invasive monitoring.

### Morbidity

Operative morbidity was graded according to the Clavien–Dindo classification (CDC)^24^, ^25^, ^26^. CDC‐0 was a normal postoperative course. CDC‐I was any deviation from the normal postoperative course that was not classified as grade II and above, and included administration of antiemetics, antipyretics, analgesics, diuretics, electrolytes and physiotherapy. CDC‐II denoted the need for pharmacological interventions not listed as CDC‐I, and included blood transfusion and total parenteral nutrition. CDC‐III was the requirement for surgical, endoscopic or radiological intervention. CDC‐IV involved life‐threatening complications requiring ICU management, and CDC‐V was death of the patient. Particular emphasis was placed on the incidence of morbidity of CDC‐III or above, defined as major morbidity.

Complications were recorded prospectively in a database, and covered postoperative inpatient stay. They were diagnosed based on clinical examination and bedside investigations, supported by radiology and microbiology assessments.

### Patient follow‐up

Patients were followed up at 2 weeks after discharge, and then at 3‐month intervals for the first year and every 6 months thereafter. At follow‐up, patients were assessed for complications and nutritional assessment. Investigations were undertaken when patients developed symptoms suggestive of recurrent disease. Surveillance was conducted for 5 years or until death, whichever occurred first^27^.

### Statistical analysis

Statistical analyses were performed using SPSS® statistics v25.0.0.0 (IBM, Armonk, New York, USA) with extension R.

Grouped data that were not normally distributed based on Shapiro–Wilk test were expressed as median (i.q.r.) values, and non‐parametric methods were used. Receiver operating characteristic (ROC) curve analyses were employed to assess the predictive value of continuous variables for the primary outcome measure and threshold, dichotomized for major morbidity as described by Youden^28^. For categorical variables, univariable and multivariable logistical regression analyses were done to identify independent associations with major morbidity. Associations found to be significant (*P* < 0·100) were retained in a binary logistic regression model using forward conditional methodology. Patient demographics were analysed between the treatment modalities by means of χ^2^ or Mann–Whitney *U* tests.

## Results

In total, 225 patients were identified who had surgery for oesophageal cancer. Twenty‐seven patients (12·0 per cent) were deemed inoperable because of local tumour invasion, and 198 underwent an anatomical resection. Of those who had a surgical resection, 106 (53·5 per cent) had a TTO (including 6 3‐stage oesophagectomies) and 92 (46·5 per cent) a THO. Twenty patients (10·1 per cent) underwent a laparoscopically assisted procedure. The median age of patients who had an oesophagectomy was 68 (i.q.r. 62–72) years, 170 (85·9 per cent) were men, and 183 (92·4 per cent) had adenocarcinoma. A total of 138 patients (69·7 per cent) underwent neoadjuvant therapy.

Some 132 patients (66·7 per cent) developed a postoperative complication, with 48 (24·2 per cent) classified as major (CDC grade III or above). Sixty‐one patients (30·8 per cent) had CDC‐0, five (2·5 per cent) had CDC‐I, 84 (42·4 per cent) had CDC‐II, 23 (11·6 per cent) had CDC‐III, 20 (10·1 per cent) had CDC‐IV, and five (2·5 per cent) had CDC‐V. The frequency of different complications that occurred in the major morbidity group is shown in *Table* *1*.

**Table 1:** Incidence of specific complications in patients with major morbidity (Clavien–Dindo grade III or above)

Complication	No. of patients (*n* = 48)
Respiratory tract infection	36 (75)
Respiratory failure	30 (63)*
Anastomotic leak	23 (48)†
Chylothorax	4 (8)
Wound infection	4 (8)
Pulmonary embolus	3 (6)

Values in parentheses are percentages.

* Resulted in one death;

† resulted in four deaths.

### Relationship between markers of systemic inflammatory response, physiological variables and Morbidity Severity Score

**Fig. 1:**
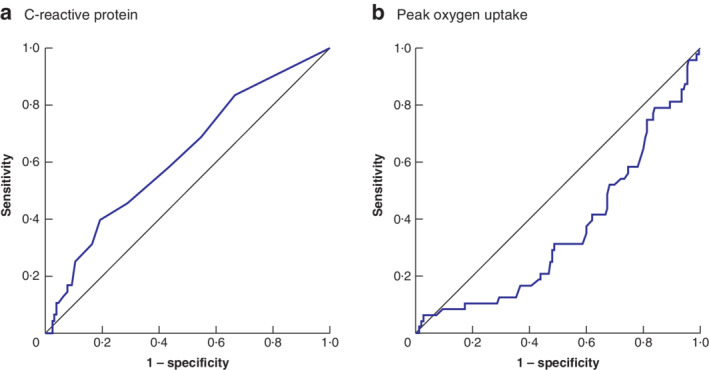
Receiver operating characteristic (ROC) curves showing predictive value of C‐reactive protein and peak oxygen uptake in major morbidity **a** C‐reactive protein; **b** peak oxygen uptake.

Baseline and area under the ROC curve (AUC) values for markers of the systemic inflammatory response and physiological variables are shown in *Table* *2*. There was no association between serum CRP and exercise testing parameters; correlation values for anaerobic threshold (*r*_s_ = −0·08, *P* = 0·232), $\dot{V}$ o_2peak_ (*r*_s_ = −0·03, *P* = 0·610) and $\dot{V}$ e/$\dot{V}$ co_2_ (*r*_s_ = −0·02, *P* = 0·754) were not statistically significant. Findings were similar for the NLR and PLR (data not shown). Using a dichotomization value of 10 mg/l, 25 (12·6 per cent) of the 198 patients had a raised CRP level. There was no difference between median measurements of $\dot{V}$ o_2peak_, anaerobic threshold or $\dot{V}$ e/$\dot{V}$ co_2_ in patients with normal or high CRP concentration respectively. The median value for CRP was 3·0 (i.q.r. 1·0–5·0) mg/l. CRP was strongly associated with major morbidity (CDC‐III or above) (AUC 0·62, 95 per cent c.i. 0·53 to 0·71; *P* = 0·012) (*Table* *2* and *Fig*. *1a*). The median value for $\dot{V}$ o_2peak_ was 20·1 (i.q.r. 17·2–24·5) ml per kg per min, and that for anaerobic threshold was 11·7 (10·1–13·8) ml per kg per min (*Table* *2*). Using the Youden index, the optimal dichotomization threshold for $\dot{V}$ o_2peak_ was 18·6 ml per kg per min (*Fig*. *1b*) and the anaerobic threshold was 11·5 ml per kg per min, with 38·4 and 47·5 per cent of patients considered to have low measurements respectively. This gave sensitivity and specificity values of 35·5 per cent and 82·8 per cent respectively for $\dot{V}$ o_2peak_, and 27·5 per cent and 79·0 per cent respectively for anaerobic threshold. Total morbidity (CDC‐I or above) rates were 73·7 per cent for low $\dot{V}$ o_2peak_ and 68·1 per cent for low anaerobic threshold respectively.

**Table 2:** Association between markers of the systemic inflammatory response, physiological variables and major morbidity

		No. of patients with level‡ (*n* = 198)				
	Median level*	Low	Normal	High	AUC†	*P* §
**Serum variables**						
Haemoglobin (g/l)	132 (121–143)	77	121	0	0·47 (0·38, 0·56)	0·555
White cell count (× 10^9^/l)	6·3 (5·1–7·7)	14	182	2	0·59 (0·49, 0·68)	0·070
Neutrophil count (× 10^9^/l)	3·8 (3·0–5·0)	10	181	7	0·56 (0·47, 0·66)	0·207
Lymphocyte count (× 10^9^/l)	1·5 (1·1–2·0)	26	165	7	0·54 (0·45, 0·64)	0·367
Platelet count (× 10^9^/l)	240 (196–291)	7	187	4	0·50 (0·41, 0·60)	0·962
C‐reactive protein (mg/l)	3·0 (1·0–5·0)	0	173	25	0·62 (0·53, 0·71)	0·012
Neutrophil/lymphocyte ratio	2·43 (1·77–3·33)				0·51 (0·42, 0·60)	0·866
Platelet/lymphocyte ratio	155 (120–215)				0·46 (0·37, 0·55)	0·364
**Cardiopulmonary exercise testing variables**						
Anaerobic threshold (ml per kg per min)	11·7 (10·1–13·8)				0·44 (0·34, 0·53)	0·194
$\dot{V}$ o_2peak_ (ml per kg per min)	20·1 (17·2–24·5)				0·36 (0·27, 0·45)	0·003
$\dot{V}$ e/$\dot{V}$ co_2_ (ml per kg per min)	30·0 (27·3–33·0)				0·60 (0·50, 0·69)	0·053

Values in parentheses are

* i.q.r. and

† 95 per cent confidence intervals.

‡ Based on local thresholds. AUC, area under the receiver operating characteristic (ROC) curve; $\dot{V}$ o_2peak_, peak oxygen uptake; $\dot{V}$ e/$\dot{V}$ co_2_, minute ventilation relative to carbon dioxide production.

§ *P* value from ROC analysis.

To adjust for potential confounders, a binary logistic regression model was developed to include the clinical factors available to the multidisciplinary team when deciding on definitive treatment (*Table* *3*). In the univariable analysis, only CRP (*P* = 0·054) and $\dot{V}$ o_2peak_ (*P* = 0·004) were associated with major morbidity. In the multivariable analysis, CRP (odds ratio (OR) 2·86, 95 per cent c.i. 1·14 to 7·17; *P* = 0·025) and $\dot{V}$ o_2peak_ (OR 2·92, 1·47 to 5·79; *P* = 0·002) were independently associated with major morbidity.

**Table 3:** Univariable and multivariable binary logistic regression analysis of preoperative factors associated with major morbidity

	Univariable analysis		Multivariable analysis	
	Odds ratio	*P*	Odds ratio	*P*
Age (< 65 *versus* 66–75 *versus* > 75 years)	1·05 (0·63, 1·76)	0·860		
Sex (F *versus* M)	1·56 (0·59, 4·35)	0·398		
Differentiation (well–moderate *versus* poor)	0·80 (0·41, 1·53)	0·493		
cTNM (1 *versus* 2 *versus* 3 *versus* 4)	0·84 (0·65, 1·08)	0·173		
Neoadjuvant therapy (no *versus* yes)	1·07 (0·53, 2·19)	0·844		
Surgical approach (TTO *versus* THO)	0·77 (0·40, 1·49)	0·444		
C‐reactive protein (normal *versus* high)	2·37 (0·99, 5·70)	0·054	2·86 (1·14, 7·17)	0·025
$\dot{V}$ o_2peak_ (< 18·6 *versus* ≥ 18·6 ml per kg per min)	2·65 (1·36, 5·15)	0·004	2·92 (1·47, 5·79)	0·002
Anaerobic threshold (< 11·5 *versus* ≥ 11·5 ml per kg per min)	1·43 (0·73, 2·77)	0·297		

Values in parentheses are 95 per cent confidence intervals. TTO, Transthoracic oesophagectomy; THO, transhiatal oesophagectomy; $\dot{V}$ o_2peak_, peak oxygen uptake.

A composite score was developed to determine whether major morbidity could be predicted with greater accuracy. This Combined Inflammatory and Physiology Score (CIPS) ranged from 0 to 2. Patients with a normal CRP level and $\dot{V}$ o_2peak_ were given a score of zero (low), a score of 1 (intermediate) was given to patients if either the CRP concentration was high or $\dot{V}$ o_2peak_ was low, and a score of 2 (high) was given to patients with both a raised CRP level and low $\dot{V}$ o_2peak_. For the 198 patients who had an anatomical resection, this resulted in 106 (53·5 per cent), 85 (42·9 per cent) and seven (3·5 per cent) patients being classified with a CIPS that was low, intermediate and high respectively. Major morbidity rates were 17·9 per cent (19 of 106), 27 per cent (23 of 85) and 86 per cent (6 of 7) in the low, intermediate and high CIPS groups respectively (*P* < 0·001). A stepwise association between increasing CIPS and major morbidity was observed. Compared with the low CIPS group, the OR was 1·70 (95 per cent c.i. 0·85 to 3·39) for the intermediate CIPS and 27·47 (3·12 to 241·69) for the high CIPS group (*P* < 0·001). The aggregate OR for CIPS and major morbidity was 2·52 (1·41 to 4·50; *P* = 0·002) and increased to 2·62 (1·42 to 4·83; *P* = 0·002) in those undergoing an open procedure. For the whole cohort of 225 patients, a higher CIPS was associated with a higher frequency of open‐and‐close procedures, affecting eight of 114 patients (7·0 per cent), 11 of 96 (11·5 per cent) and eight of 15 (53 per cent) in the low, intermediate and high CIPS groups respectively (*P* < 0·001).

## Discussion

Metabolic measures of the SIR together with physiological measures of cardiorespiratory fitness ($\dot{V}$ o_2peak_) were independently associated with major complications in patients selected to undergo curative oesophagectomy for adenocarcinoma. Major operative morbidity was threefold higher in patients with an increased CRP concentration and poor cardiorespiratory fitness. Combining these parameters established a novel composite risk score (CIPS), from which six of seven patients (86 per cent) with a score of 2 developed major morbidity compared with 19 of 106 (17·9 per cent) with a score of zero.

Previous reports^4^, ^29^ have contended that the SIR is closely associated with postoperative complications in colorectal cancer, and cardiovascular disease, diabetes, poor diet, obesity and smoking have all been associated with increased CRP levels and poorer prognosis.

The present findings raise the possibility that a programme of prehabilitation combined with measures to attenuate the SIR might reduce perioperative complications. A randomized trial of prehabilitation in elective major abdominal surgery^30^ showed that prehabilitation reduced postoperative complications by 51 per cent, and another randomized study^31^ showed that prehabilitation was associated with higher functional capacity before surgery (mean(s.d.) 6‐min walk distance change 36·9(51·4) m *versus* −22·8(52·5) m for no prehabilitation; *P* < 0·001), which was maintained into the postoperative period (15·4(65·6) *versus* −81·8(87·0) m respectively; *P* < 0·001).

Using a prehabilitation programme to improve physiological performance and potentially attenuate the SIR thus seems an attractive proposition, although how best to do this and understanding which patients will gain the most benefit are unresolved issues. Incorporating anti‐inflammatory medication into a prehabilitation care package is not without risk. A meta‐analysis^32^ of non‐steroidal anti‐inflammatory use in colorectal surgery suggested an increased risk of anastomotic leak (OR 1·96). A similar finding was also observed in patients undergoing oesophagogastrectomy (OR 5·24)^33^. Conversely, two doses of perioperative dexamethasone reduced the postoperative inflammatory response and complication rate in patients undergoing colectomy for cancer^34^.

The results of the present study merit validation in an independent cohort, although limitations in its methodology mean that these findings must be interpreted with caution. The patient cohort was a highly selected group with adenocarcinoma or squamous cell carcinoma, most of whom had neoadjuvant therapy and surgery, so were not representative of all patients diagnosed with oesophageal cancer who undergo operation. Data relating to blood loss and duration of surgery were not collected, and these may be important confounders. This analysis did not include patients who had an open‐and‐close procedure. Twenty patients had a laparoscopically assisted oesophagectomy, and the CIPS will therefore require validation in this subgroup. The study, however, has several strengths. Patients were recruited from a consecutive series of patients diagnosed with oesophageal cancer, from a single UK geographical region, all treated by the same specialist team, using a standardized staging algorithm and team‐based operative techniques, with international audited and published quality control^35^.

Combining CRP level and $\dot{V}$ o_2peak_ into a novel prognostic score has disclosed a subset of patients at high risk of developing complications. Refining cardiopulmonary fitness by using a multimodal prehabilitation treatment bundle that might attenuate the SIR and reduce postoperative morbidity seems an attractive approach for patients undergoing oesphagectomy for cancer.

## Collaborators

Members of the South‐East Wales Oesophagogastric Cancer Collaborative include: B. Bahlmann, R. Barlow, G. Blackshaw, A. Christian, G. Clark, X. Escofet, A. Foliaki, T. Havard, M. Henwood, S. A. Roberts, A. Willicombe, J. Witherspoon.
